# Selenium incorporation using recombinant techniques

**DOI:** 10.1107/S0907444909038207

**Published:** 2010-03-24

**Authors:** Helen Walden

**Affiliations:** a Protein Structure and Function Laboratory, Cancer Research UK London Research Institute, Lincoln’s Inn Fields, London WC2A 3PX, England

**Keywords:** selenium incorporation, selenium labelling

## Abstract

Using selenomethionine to phase macromolecular structures is common practice in structure determination, along with the use of selenocysteine. Selenium is consequently the most commonly used heavy atom for MAD. In addition to the well established recombinant techniques for the incorporation of selenium in pro­karyal expression systems, there have been recent advances in selenium labelling in eukaryal expression, which will be discussed. Tips and things to consider for the purification and crystallization of seleno-labelled proteins are also included.

## Abstract 2

An overview of techniques for recombinant incorporation of selenium and subsequent purification and crystallization of the resulting labelled protein.

## 1. Introduction

**Figure 1:**
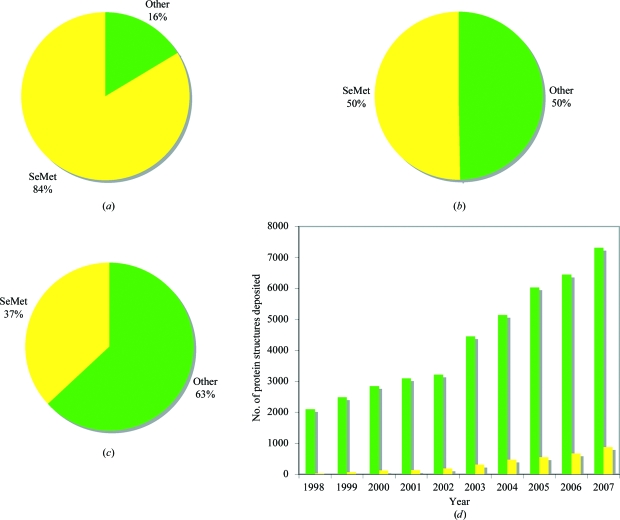
Incidences of SeMet use for phasing in the Protein Data Bank (PDB). (*a*) Pie chart showing the proportion of MAD-phased structures using SeMet (yellow) as a percentage of the total structures phased using MAD. (*b*) Proportion of SAD-phased structures using SeMet (yellow) as a percentage of the total SAD-phased structures. (*c*) Proportion of structural genomics (SGX) derived crystal structures solved using SeMet (yellow) as a percentage of the total number of SGX-derived crystal structures. (*d*) Total number of crystal structures deposited each year (green), with the number solved using SeMet shown in yellow. Numbers were correct at the end of 2008 for (*a*)–(*c*) and the end of 2007 for (*d*).

In 1957, Cowie and Cohen demonstrated the ability of *Escherichia coli* to incorporate selenium instead of sulfur during protein biosynthesis (Cowie & Cohen, 1957 ▶). Several decades later, this trick was exploited in the development of powerful phasing techniques to solve the phase problem in macromolecular crystallography (Hendrickson *et al.*, 1990 ▶). Hendrickson first reported the possibility of using selenomethionine (SeMet) as a phasing tool in 1990 and the ease of incorporating selenium in recombinant systems has led to selenium being the first choice for multiple-wavelength (and single-wavelength) anomalous dispersion experiments (Figs. 1 ▶ *a* and 1 ▶ *b*). Incorporating SeMet recombinantly produces modified protein without the structural disturbance commonly associated with heavy-atom incorporation and removes the need for time-consuming and challenging screening for heavy-atom derivatives.

Since the advent of structural genomics (SG) and with the increasing output from SG consortia, it has become clear that SeMet labelling for structure determination is making a profound difference to structure determination (Figs. 1 ▶ *c* and 1 ▶ *d*). In addition to being a powerful phasing tool, SeMet substitution is also useful for sequence assignment and chain tracing in crystallography. In some cases, it has been reported that SeMet-derived crystals diffracted to a higher resolution than the native counterpart (Koon *et al.*, 2004 ▶; Li *et al.*, 2008 ▶; Quevillon-Cheruel *et al.*, 2004 ▶). In prokaryal systems incorporation of SeMet is often close to 100%, particularly when a methionine-auxotrophic strain such as B834 is used (Leahy *et al.*, 1992 ▶). However, some proteins are just not amenable to production in bacteria and therefore in more recent years the incorporation of selenium has been adapted from simple prokaryotic expression systems to eukaryal systems. There have been a number of successful cases of incorporation in eukaryotic systems, including yeast, insect cells and mammalian cells (Larsson *et al.*, 2002 ▶; Lustbader *et al.*, 1995 ▶; Bellizzi *et al.*, 1999 ▶). Given that the overall cost of structure determination has been estimated at more than £100 000 per structure (Chandonia & Brenner, 2006 ▶), the additional costs associated with use of SeMet are relatively small and can be reduced by using a dl-SeMet mixture at double the concentration of the more expensive l-SeMet. The only real drawback of recombinant SeMet incorporation is yield of labelled protein. This article will outline some of the considerations for each system and discuss methods for checking incorporation and crystallization of the resulting protein.

## 2. Prokaryotic systems

By far the simplest and the most widely used system for the incorporation of selenium is prokaryal expression. The initial reports of SeMet labelling of recombinant proteins entailed growing a methionine-auxotrophic strain of *E. coli* (DL41) in minimal media supplemented with amino acids and SeMet (Hendrickson *et al.*, 1990 ▶). This is a very successful approach, resulting in close to 100% Se substitution, and is often used for structural problems, typically using the BL21-derived auxotrophic strain B834 (DE3) available from Invitrogen, Novagen, Stratagene and others. However, there are some disadvantages to the method as the yield of protein is often only 15–20% of that of the native protein. Also, growing *E. coli* in minimal media is time-consuming as it will often take up to 24 h for cultures to reach a cell density of OD_600nm_ = 0.6. However, non-auxotrophic strains can be used to reliably incorporate selenium, albeit slightly less efficiently (Van Duyne *et al.*, 1993 ▶; Doublié, 1997 ▶, 2007 ▶). If methionine bio­synthesis is inhibited shortly before induction, >90% SeMet incorporation can be observed. Inhibition of Met bio­synthesis can be achieved by the use of high concentrations of iso­leucine, leucine, phenylalanine, lysine and threonine, which are added to the culture shortly (∼10–15 min) before induction (for recipes, see Doublié, 2007 ▶, and the end of this review). The yield of protein is often better than with the auxotroph, the cell density is greater and the growth levels are closer to those obtained using LB for native growth.

Another effective means of achieving SeMet incorporation is to grow the culture directly in defined LeMaster media without any requirement for methionine-biosynthesis inhibition (LeMaster & Richards, 1985 ▶; Bravo *et al.*, 1998 ▶). This method also leads to good growth and yields and high incorporation rates. It is also less involved in terms of media preparation.

In addition, it has recently been reported that the use of auto-inducing media with a non-auxotrophic strain is also a viable system for producing SeMet-labelled proteins (Studier, 2005 ▶). This system entails the transformation of cells and then culturing them in a smaller volume for greater aeration for a total of ∼24 h at a culture temperature of 310 K (increasing to 40 h or longer at lower growth temperatures such as 298 or 289 K; Sreenath *et al.*, 2005 ▶; Doublié, 2007 ▶). This method generally leads to >90% SeMet incorporation and has the advantage of high cell density and good recovery of protein. The dis­advantages are the costs and preparation associated with the auto-inducing media and the length of time required for culture growth.

In short, the decision as to which experiment to execute depends on how much SeMet incorporation is required (*i.e.* how many ordered sites for substitution are predicted? Would only 90% incorporation be sufficient?), time constraints on the cell preparation (*e.g.* proximity of the next synchrotron trip!) and considerations for yield (*i.e.* how well the protein is expressed).

Table 1 ▶ summarizes the characteristics of each system. There are many excellent protocols for the expression of SeMet proteins in prokaryotic systems (Doublié, 1997 ▶, 2007 ▶) and a short set of notes is included at the end of this review.

## 3. Eukaryotic systems

### 3.1. Baculovirus-infected insect-cell expression

The most commonly used alternative to *E. coli* for the production of soluble recombinant protein is baculovirus-infected insect-cell expression (Possee, 1997 ▶). The advantages of this system include the incorporation of post-translational modifications such as phosphorylation and glycosylation and the greater solubility of some eukaryal proteins.

There are many examples of SeMet incorporation in insect cells for both secreted and intracellularly expressed proteins (Bellizzi *et al.*, 1999 ▶; Fremont *et al.*, 1998 ▶; Aricescu, Assenberg *et al.*, 2006 ▶; McWhirter *et al.*, 1999 ▶). In general, secreted proteins have a higher incorporation of SeMet than intracellularly expressed proteins. This is because when the Met-containing medium is exchanged for SeMet medium, any unlabelled protein that has already been secreted is removed. However, in 2007 Cronin and coworkers reported a systematic study of SeMet-labelling proteins in baculovirus expression-vector systems in order to determine an optimal protocol for obtaining consistent and reliable SeMet-labelled protein for both intracellularly expressed and secreted proteins (Cronin *et al.*, 2007 ▶). They found that the time of addition of SeMet was crucial for incorporation.

To summarize, SeMet should be added within the first 16 h post-infection for optimal substitution and there is a trade-off between the SeMet concentration in the medium and the yield of protein and level of SeMet incorporation. As with the situation in prokarya, the experimental decision depends on how much SeMet incorporation is required and considerations for yield. Table 2 ▶ summarizes these findings and a basic protocol for insect-cell expression is listed at the end of this review.

### 3.2. Yeast

As an alternative to prokaryal systems, yeast is an attractive host as it is relatively inexpensive and simple to culture, often yielding 2 mg of purified unlabelled protein per litre of cell culture (Martzen *et al.*, 1999 ▶; Gelperin *et al.*, 2005 ▶). There are also examples of the production of SeMet-labelled proteins  in both *Pichia pastoris* and *Saccharomyces cerevisiae* (Worthylake *et al.*, 1998 ▶; Macbeth *et al.*, 2005 ▶; Jidenko *et al.*, 2005 ▶; Bushnell *et al.*, 2001 ▶; Larsson *et al.*, 2002 ▶). However, the drawback to the system has been the cytotoxicity of SeMet and although some successful reports have been published, the incorporation of selenium has hovered around the 50% mark. This was not sufficient to phase the RNA polymerase II complex, but proved useful for chain tracing (Bushnell *et al.*, 2001 ▶). Recently, Malkowski and coworkers have reported that blocking *S*-adenosylmethionine (SAM) biosynthesis allows higher SeMet incorporation (>95%), following their initial hypothesis that the reason for the toxicity of selenium is the conversion of SAM to the seleno-derivative (Malkowski *et al.*, 2007 ▶). Consequently, they developed an SeMet-resistant strain of *S. cerevisiae* which should prove useful for structural studies. Their initial reports suggest yields of SeMet-labelled protein of between 30 and 60% of that obtained in un­labelled expression. Finally, although not a recombinant technique *per se*, the complete replacement of Met with SeMet has recently been reported in yeast by growing yeast entirely on SeMet, suggesting a viable approach for purifying SeMet-labelled yeast proteins from source (Ouerdane & Mester, 2008 ▶).

### 3.3. Mammalian

For production, some proteins require conditions that are as close to native as possible. In such cases, stably transfected mammalian cell lines are used as expression hosts for the protein of interest. In individual cases, these hosts have been adapted to produce SeMet-labelled proteins. For example, Chinese Hamster Ovary (CHO) cells were used to achieve 93% SeMet incorporation for a secreted protein (Lustbader *et al.*, 1995 ▶), while HEK293 cells achieved 60% incorporation for a secreted protein (Aricescu, Lu *et al.*, 2006 ▶). In 2006, Barton and coworkers described a method for efficient SeMet labelling in mammalian cells, which entailed depleting the medium of methionine for 12 h prior to addition of SeMet at 60 mg l^−1^ and culturing for a further 48 h for roller-bottle cultures or for 72 h for dishes. In their test cases, this yielded 60–80% of the amount of protein produced during unlabelled expression with greater than 90% SeMet incorporation (Barton *et al.*, 2006 ▶).

In addition to the prokaryal and eukaryal systems reviewed above, the use of cell-free synthesis has been reported as an alternative (Kigawa *et al.*, 2002 ▶) and, impressively, the labelling of an entire virus by finding a suitable host has recently been reported (Kivela *et al.*, 2008 ▶). These advances suggest that the use of selenium for structure determination is only set to increase.

## 4. Considerations

### 4.1. Oxidation state

It has been reported that oxidized SeMet has an increased magnitude in its absorption edge compared with reduced SeMet (Smith & Thompson, 1998 ▶). Subsequently, several groups have exploited deliberate oxidation of selenium in order to maximize the anomalous signal. The structure of the TolC receptor was solved after treatment of the protein with 0.1% hydrogen peroxide for 10 s, although this would not be a useful treatment for the majority of enzymes (Sharff *et al.*, 2000 ▶). Thomazeau and coworkers reported the phasing of threonine synthase using oxidized selenium, in which peroxide was not employed (Thomazeau *et al.*, 2001 ▶). Certainly, this is something that is worth considering if there are few or dis­ordered methionines in the crystal. However, a note of caution is that an oxidized SeMet would be expected to be more radiation-sensitive than its reduced form, radiation-induced decay of the anomalous signal being one of the problems in using selenium as a phasing atom (Holton, 2007 ▶). Including a strong reducing agent such as tris(2-carboxy­ethyl)phosphine (TCEP) to push the SeMet back into a reduced state is also useful. The important point is that the state of the SeMet should be uniform, otherwise the Se *K* edge moves to a higher energy, smudging the edge.

### 4.2. Occupancy

The purpose of recombinantly incorporating heavy atoms is for eventual structure determination. For this to be successful, the anomalous scatterer needs to provide sufficient phasing power. Predicting the strength of an anomalous signal can be useful in deciding what levels of incorporation of heavy atom will be minimally required for a successful phasing experiment (Garman & Murray, 2003 ▶; http://skuld.bmsc.washington.edu/scatter/AS_signal.html). In general, the heavier the atom the larger the signal and the easier it will be to measure. As selenium is actually fairly small by heavy-atom standards, it is important to maximize the phasing power. This can be achieved through optimum occupancy. Occupancy is defined as the fraction of unit cells in a crystal that contain the atom in question. If occupancy is low it may be difficult to locate the heavy atom because of noise in the map resulting from approximations inherent in the data. This inevitably reduces the phasing power of the heavy atom and in the case of the RNA polymerase II complex expressed in yeast a 50% incorporation of SeMet was not sufficient to phase the structure (Bushnell *et al.*, 2001 ▶). Thus, ensuring maximum incorporation of selenium will provide better phasing power for structure determination.

### 4.3. Not enough methionines?

Although the majority of proteins contain methionine (estimated at 80%; Boggon & Shapiro, 2000 ▶), for SeMet labelling to be sufficient for phasing the substructure there must be enough ordered sites. If the protein in question has a low level of methionine content, there are strategies that can be employed to maximize the phasing power of the experiment. Firstly, at the DNA level, extra methionines can be engineered in. Leucine and isoleucine are good candidates for substitution given their hydrophobic nature and their likelihood of being ordered in the core of the protein. Homologues, even remote ones, can provide a useful guide to engineering in methionines. This strategy can also prove useful if sequence assignment is problematic.

Secondly, double labelling of the protein by using selenocysteine in combination with SeMet can be employed (Strub *et al.*, 2003 ▶). This increases the cost of the experiment and can also limit the yield, but does allow the production of more anomalous atoms in the crystal.

Finally, another option is to use the larger, although significantly less stable, analogue telluromethionine. When successful, this substitution provides a strong anomalous signal and therefore significant phasing power (Budisa *et al.*, 1997 ▶).

## 5. Measuring the incorporation of selenium

After the efforts expended to achieve SeMet incorporation, it is essential to verify that is has occurred prior to crystallization and data collection. There are several methods by which to check the substitution of Met by SeMet (and Cys by SeCys). Mass spectrometry is the primary technique and is very effective. It can be used for peptide-fragment analysis to measure the difference in mass between the peptides yielded from tryptic digests of unlabelled and labelled protein. Intact mass analysis is also sensitive enough to measure the difference in mass between labelled and unlabelled protein and can also give an idea as to the oxidation state of the seleniums in solution. In the rare cases where a protein does not fly well in the mass-spectrometry experiment, quantitative amino-acid analysis can be used to measure the loss of methionine, with the assumption that it has been replaced by SeMet. MicroPIXE (proton-induced X-ray emission) is another useful quantitative tool for the measurement of selenium content in a protein sample (Garman, 1999 ▶). A fluorescence scan of the protein either in solution or in the crystalline form will also detect selenium in the sample and most modern beamlines incorporate the fluorescence emission spectrum as a matter of a course, revealing the elemental composition of the sample.

## 6. Crystallization

One of the major advantages of recombinantly introducing an anomalous scatterer into a protein is the fact that the resulting crystal will be used to obtain all data, making the heavy-atom derivative isomorphous with the protein structure. In the majority of cases, crystallization of an SeMet-labelled protein occurs under identical or very similar conditions to the native protein. If sufficient material is obtained, then re-screening for crystallization conditions is advisable as there are instances in which better diffracting crystals or new crystal forms have been obtained using the labelled protein. If this is not successful, cross-seeding with the unlabelled crystals is a very useful technique (see, for example, Love *et al.*, 2003 ▶) and is especially so when the SeMet-labelled protein yields are low. In general, SeMet-substituted proteins tend to be a little less soluble than their native counterparts owing to increased hydrophobicity and therefore lowering the protein or precipitant concentration is a good place to start if identical conditions do not yield crystals. The crystals themselves are often slightly less stable than the native crystals, probably owing to the sensitivity of selenium to oxidation compared with sulfur. Cryocooling the crystals as soon as they have grown is one way around this problem and adding a strong reducing agent, even at the point of mounting the crystal (providing it survives), will also aid the stability.

## 7. Conclusions

In summary, the use of recombinant SeMet labelling for structure determination was a huge step forward in experimental phasing and is now a commonly used technique. The advances in expression systems outlined above have made it an even more attractive technique for phasing, and the numbers of SeMet MAD/SAD experimentally phased structures is only going to increase. There are many useful recipes and protocols for SeMet (and SeCys) labelling of proteins (Barton *et al.*, 2006 ▶; Kivela *et al.*, 2008 ▶; Cronin *et al.*, 2007 ▶; Strub *et al.*, 2003 ▶; Doublié, 2007 ▶, 1997 ▶); listed below are some notes to get you started.

## 8. Materials and methods

### 8.1. Prokaryal expression using a methionine auxotroph

Transform B834 (DE3) cells (Invitrogen, Stratagene, Novagen) with expression vector.

Grow overnight culture in LB media at 310 K, shaking at 150–200 rev min^−1^.

Spin down cells gently (2000*g*) and wash the pelleted cells with M9 minimal media (for recipes, see Doublié, 2007 ▶).

Inoculate 1 l culture(s) of M9 minimal media containing appropriate antibiotics, 5–10 g l^−1^ glucose, all amino acids except Met at 40 mg l^−1^, l-SeMet at 50 mg l^−1^, 2 mg l^−1^ thiamine and 2 mg l^−1^ biotin.

Induce as for native.

Harvest as for native.

Expect the time taken to reach mid-log phase to be 12–18 h.

When purifying the protein, degas all buffers (unless aiming for deliberate selenium oxidation) and add a reducing agent such as dithiothreitol, β-mercaptoethanol or tris(2-carboxy­ethyl)phosphine (TCEP).

### 8.2. Prokaryal expression inhibiting methionine biosynthesis

Grow overnight culture in LB medium at 310 K, shaking at 150–200 rev min^−1^.

Spin down cells gently (2000*g*) and wash the pelleted cells with M9 minimal media.

Inoculate 1 l culture(s) of M9 minimal medium containing appropriate antibiotics and 5 g l^−1^ glucose.

At mid-log phase add 100 mg l^−1^ each of phenylalanine, lysine and threonine, 50 mg l^−1^ each of leucine, isoleucine, valine and l-SeMet.

Induce 15 min after addition of amino acids.

Harvest as for native.

Purify with degassed buffers and reducing agent.

### 8.3. Prokaryal expression in LeMaster medium

Grow overnight culture in LB medium at 310 K, shaking at 150–200 rev min^−1^.

Inoculate 1 l culture(s) of defined LeMaster medium (LeMaster & Richards, 1985 ▶) containing appropriate antibiotics.

Induce as for native.

Harvest as for native.

Purify with degassed buffers and reducing agent.

### 8.4. Prokaryal expression using auto-inducing media

See Studier (2005 ▶), Sreenath *et al.* (2005 ▶) and Doublié (2007 ▶) for protocols using autoinduction media.

Harvest as for native.

Purify with degassed buffers and reducing agent.

### 8.5. Eukaryal expression in insect cells

Infect cells in methionine-containing media.

16 h post-infection, change media to methionine-free media and supplement with 50–200 mg l^−1^ (see Table 2 ▶) l-­SeMet.

Culture for 48 h post-infection.

Harvest as for native.

Purify with degassed buffers and reducing agent.

## Figures and Tables

**Table 1:** Comparison of methods for prokaryal expression of SeMet-labelled protein

Met auxotroph, B834	Met inhibition, BL21	No Met inhibition, defined LeMaster medium + SeMet, BL21	Auto-induction, BL21
Slow growth, longer lag phase, low cell density	Closer to LB levels of growth, good cell density	Close to LB levels of growth, good cell density	High cell density, ease of culture, good growth; takes a long time (48 h)
Poor yields, ∼20% that of unlabelled	OK yields, ∼30–80% that of unlabelled	Good yields, ∼50–70% that of unlabelled	Good recovery of protein, often better than induced unlabelled
∼100% incorporation of SeMet	>90% incorporation of SeMet	>90% incorporation of SeMet	>90% incorporation of SeMet

**Table 2:** Comparison of strategies for insect-cell expression of SeMet-labelled protein

SeMet (mg l^−1^)	Yield (% that of unlabelled expression)	Incorporation of SeMet (%)
50–100	∼50	∼50
200	∼20	>70
